# The Status Quo and Attribution of Wildlife Crimes: A Study of Cases in China From the Perspective of Ecological Economic Ethics

**DOI:** 10.3389/fpubh.2021.751103

**Published:** 2021-10-29

**Authors:** Zhongmin Zhang, Yuting Zeng, Danqi Xie

**Affiliations:** ^1^Law School, Institute of Ecological Civilization, Zhongnan University of Economics and Law, Wuhan, China; ^2^Law School, Zhongnan University of Economics and Law, Wuhan, China; ^3^School of Philosophy, Institute of Economic Ethics, Zhongnan University of Economics and Law, Wuhan, China

**Keywords:** COVID-19 epidemic, wildlife crimes, economic ethics, ecological economic ethics, public health

## Abstract

The COVID-19 pandemic, which has ravaged the world, has led to a rethinking of the relationship between humans and nature and the clichés of the economic-centered model. Thus, the ecological economy has been reviewed, especially from an ethical worldview. This paper uses statistical methods to retrieve and categorize 3,646 wildlife crime cases for analysis and quantitative research. It adopts legal and ethical perspectives to analyze the subject and the subjective, incidence, and sentencing factors of wildlife crimes and uses the ecological economic ethical model to measure wildlife crimes. We argue that the existing judicial system fails to answer the difficulties of the economic ethics of wildlife crimes. It is recommended that ecological and economic ethical awareness be internalized. We suggest calling for comprehensive legislation on wildlife crimes from the perspective of ecological economic ethics to effectively prevent and reduce wildlife crime and eventually promote public health.

## Introduction

As COVID-19 affects the world, wildlife conservation issues have returned to the limelight. COVID-19 is much more serious than SARS's shock in 2002–2003. Notably, the COVID-19 pandemic brought tragic consequences for public health in both 2020 and 2021. According to the World Health Organization, COVID-19 is the disease caused by a new coronavirus called SARS-CoV-2. Moreover, most coronaviruses originate from animals (1). The World Organization for Animal Health confirms that the virus that triggered the outbreak originated from animals. Research since the COVID-19 pandemic began has shown that a range of animals, including wild and farmed species, are susceptible to infection (2). This ecological alarm and public health crisis are a reminder for all of humanity, and the outbreak of the COVID-19 pandemic has led to a review of the relationship between humans and nature, especially the relationship between human beings and animals. Eventually, this phenomenon has encouraged society to become more introspective about solutions to the ecological crisis.

A crime, as a damaging and destructive act against human society, involves conduct that is incompatible with social expectations. Crime is viewed not only as harm to a community's recognized morals and sentiments but also as a violation of others' rights and the order of the legal system. Crime is contrary to the public interest and harmful to individuals or public safety (3). Engels classically noted that crime is the most extreme manifestation of contempt for the social system (4). The most typical wildlife crimes are among the top five environmental crimes, including the illegal hunting and killing of rare and endangered wildlife and the illegal purchase, transportation, and sale of rare and endangered wildlife and their manufactured products (5). This is a testimony to offenders' disdain for the wildlife conservation order because the violation and denial of wildlife rights are the most typical misconduct. Therefore, we raise the following questions: what type of regularity does the subject of wildlife crimes present? What is its subjective element? Is it based on certain values, and does it reflect a certain social consciousness? What are the objective patterns of wildlife crimes? What values can we use for judgment? Can justice, as the last safeguarding frontier of wildlife protection, provide a full remedy? This paper focuses on wildlife crimes, conducts an in-depth analysis of their causes and contributes to restoring the order of wildlife protection.

## Materials and Methods

### Focusing on the Integration of Three Disciplines

We used “wildlife crime” as a keyword to search on the website “www.pkulaw.com” (6) and obtained detailed research data. This database collects many repeated cases; thus, we removed repeated cases, such as second trial cases and retrial cases, and chose criminal and first trial cases because they echoed our goals. In the end, we obtained 3,646 cases that met our criteria, namely, the first trial criminal cases with no repeated cases. Then, we used statistical methods to analyze the variables of wildlife crimes, including the subject and the subjective, incidence, and sentencing variables. Next, we classified these data and transformed some of the textual data into numeric data. The data are shown in the form of statistical graphs that characteristically display the variable distributions and reveal the structure, inner relations of the cases, and trends of the variables. From legal and ethical perspectives, our research can help to integrate the three disciplines of statistical analysis and processing, legal logic, and ethical thinking.

### A New Study Pattern

A traditional paradigm of crime research is to address the phenomenon of crime, analyze the causes of crime, and then propose solutions; this approach analyzes the crime situation (incidence, type of dispute, means and forms, areas, consequences, and characteristics) and basic situation of the perpetrators (age, educational background, occupation, and criminal history) to summarize the causes of the crime (social and personal causes) and form crime prevention countermeasures (7). For instance, when we analyze a criminal case, we consider the objective factors first such as the answers to who committed the crime? What did he or she do? What were the results? Is there causality between the behaviors and the results? Sequentially, we identify the subjective factors, specifically, intentional crime or negligent crime and the age and capability of the perpetrator. This is how criminologists conduct their research on crimes of various types.

This paper adopts a new research paradigm that aims to derive the inherent rules of cases through empirical research on a large number of cases. This paper concentrates on ethical thinking integrated with legal analysis and develops an innovative pattern that compares and contrasts the subject and the object and the subjective and objective factors of different wildlife crimes to consider social awareness, value judgments, and their ranks. Thus, the width and depth of ethical misconduct can be identified in all aspects. The innovation of this paper is that it moves away from traditional perspectives and uses empirical research through case studies, ethical value judgments, and a jurisprudential normative analysis. An innovative model that crosses over the disciplines of statistics, law, and ethics is designed to study the ethical reasons for wildlife crimes and the absence of public health.

## Results

The website “www.pkulaw.com” was used as a search platform to collect “wildlife crime” cases. The trial procedure was limited to “trial at first instance” and “criminal cases” were input as the type of dispute. The final retrieval of 3,646 articles was achieved after eliminating duplicate and unrelated cases. The cases were as follows: “illegal hunting and killing of rare and endangered wild animals;” “illegal purchase, transportation, and sale of rare and endangered wild animals and their manufactured products;” “illegal hunting;” and “smuggling of rare animals, illegal transportation, and sale of rare and endangered wild animals.” Among the cases (Table 1), there were 1,287 cases of “illegal hunting and killing of rare and endangered wild animals,” 1,894 cases of “illegal purchase, transportation, and sale of rare and endangered wild animals and their manufactured products,” 537 “illegal hunting” cases, and 10 cases of “smuggling of rare animals, illegal transportation, and sale of rare and endangered wild animals.” Notably, in the latest revision of China's Criminal Law in 2020, a new clause was added under Article 341 to criminalize the hunting, purchasing, transporting, and selling of rare and endangered wild animals criminalize hunting, purchasing, transporting, and selling—for the purpose of consumption as food. However, this crime was newly updated, and relative crimes were rare. We did not take this crime into consideration. We can conclude that “illegal hunting and killing of rare and endangered wild animals” and “illegal purchase, transportation and sale of rare and endangered wild animals and their manufactured products” account for the largest number of cases.

**Table 1:** Wildlife crimes distribution.

**Crimes**	**Number**	**Proportion (%)**
Illegal hunting and killing of rare and endangered wild animals	1,287	34.52
Illegal purchase, transportation, and sale of rare and endangered wild animals and their manufactured products	1,894	50.80
Illegal hunting	537	14.40
Smuggling of rare animals, illegal transportation, and sale of rare and endangered wild animals	10	0.27
Total	3,728	100

### Subjective Aspects

The subjects of the crime were mainly natural persons in 3,593 cases, which accounted for 98.57% of the total number of wildlife crimes. In contrast, unit crimes constituted a total of 53 cases, which accounted for only 1.45%. Among the wildlife crimes committed by natural persons, 501 cases were committed by minors (under 18 years old), which accounted for 13.94% of the total.

Of all wildlife crime cases (Table 2), 1,295 cases were first offenses, and 693 were second-time or greater offenses, and they constituted 35.51 and 19.00%, respectively. The numbers of cases in which the subject of the crime surrendered and demonstrated repentance were 2,574 and 2,307, respectively, which represented 70.60 and 63.27% of the total, respectively. These numbers suggest a higher degree of first-time offending and a certain degree of repentance on the part of the criminal subjects.

**Table 2:** The subjective aspects (negligence crime).

**Circumstance**	**Number**	**Proportion (%)**
First offense	1,295	35.51
Second-time or greater	693	19.00
Surrendering criminal	2,574	70.60
Demonstrate repentance	2,307	63.27

In addition, 908 cases were intentional crimes, and 1,314 offenders had criminal records (Table 3), which constituted 24.90 and 36.04%, respectively. There were 502 cases where the perpetrator was armed with a gun, which was 13.77% of the total. The defendants were represented by a lawyer in 1,984 of the 3,646 cases, or 54.42% of the total number. There were 302 cases of attempted crimes or 8.28% of the total. This number is higher than that of first-time and occasional offenders and indicates a lower level of remorse for crimes and a higher likelihood of recidivism.

**Table 3:** The subjective aspects (intentional crime).

**Circumstance**	**Number**	**Proportion (%)**
Intentional crime	908	24.90
Criminal record	1,314	36.04
Gun-toting	502	13.77

### Sentencing

As shown in Table 4, we collected 3,646 wildlife crime cases and identified 4,721 defendants, 1,321 of whom were given suspended sentences, which accounted for 51% of the number of cases with fixed-term imprisonment. The individuals who were sentenced were placed on between a minimum 3-year probation and a maximum of 6 years and 3 months of probation. A total of 58,463,275 RMB in fines and confiscated properties was imposed, which is an average of 12,383.66 RMB per defendant and a minimum value of 500 RMB and a maximum of 1 million RMB. Among the defendants, 2,590 were sentenced to fixed-term imprisonment, which constituted 54.86% of the total number, with a minimum term of 2 months and a maximum term of 18 years. The set-term imprisonment period was mainly within 3 years. The distribution of the sentencing periods is shown in Table 5.

**Table 4:** Punishments and circumstances.

	**Consequences**	**Number**	**Proportion (%)**
Punishments	Detention	2,107	44.63
	Community correction	673	14.26
	Suspended sentence	1,321	27.98
	Sentence of more than 10 years	150	3.18
	Combined sentence of principal and supplementary punishment	3,201	67.80
	Sentence of “not guilty”	6	0.13
Circumstances	Lesser punishment	2,515	53.27
	Commutation	1,789	37.89
	Abatement of criminal punishment	737	15.61
	Heavier punishment	391	8.28

**Table 5:** The distribution of the sentencing period.

**Period**	**Number**	**Proportion (%)**
0–1 Year	603	23.28
1 Year	404	15.60
1–2 Years	309	11.93
2 Years	299	11.54
2–3 Years	125	4.82
3 Years	223	8.61
3–4 Years	39	1.51
4 Years	34	1.31
4–5 Years	9	0.35
5 Years	155	5.98
5–6 Years	50	1.93
6 Years	70	2.70
6–7 Years	13	0.51
7 Years	48	1.85
7–8 Years	5	0.19
8 Years	26	1.00
8–9 Years	0	0.00
9 Years	16	0.62
9–10 Years	1	0.04
10 Years	78	3.01
Above 10 Years	83	3.20
Total	2,590	100

The distributions of the sentencing periods within 1 year were concentrated at 6, 8, 10 months, and 1 year. The sentencing times were concentrated mainly within 3 years. It can be concluded that the sentence (penalty) is lighter than the serious consequences of the offenders' crimes. Judges prefer less punishment, commutation, and the abatement of criminal punishment to heavier punishment. People charged with illegal hunting misconduct were punished only with an additional fine. Therefore, light penalties and suspended sentences are prominent in wildlife crimes. Although the sentencing criteria range from <5 years to more than 10 years of imprisonment, the statutory penalty for wildlife crimes cannot cover different circumstances, and the term limit of a statutory sentence is relatively low compared with other environmental pollution offenses. Thus, the punitive function and deterrent effect of the penalty are limited (8). In practice, scholars and experts also tend to endorse lighter sentences for wildlife crimes and call for reduced punishment, mitigation and acquittal for wildlife crimes. It can be concluded that judges, experts, and scholars are not sufficiently aware of wildlife protection to reach an ecological economic ethical consensus within the domain of the legal community. Therefore, judicial remedies for wildlife are weakened.

### The Years

**Figure 1:**
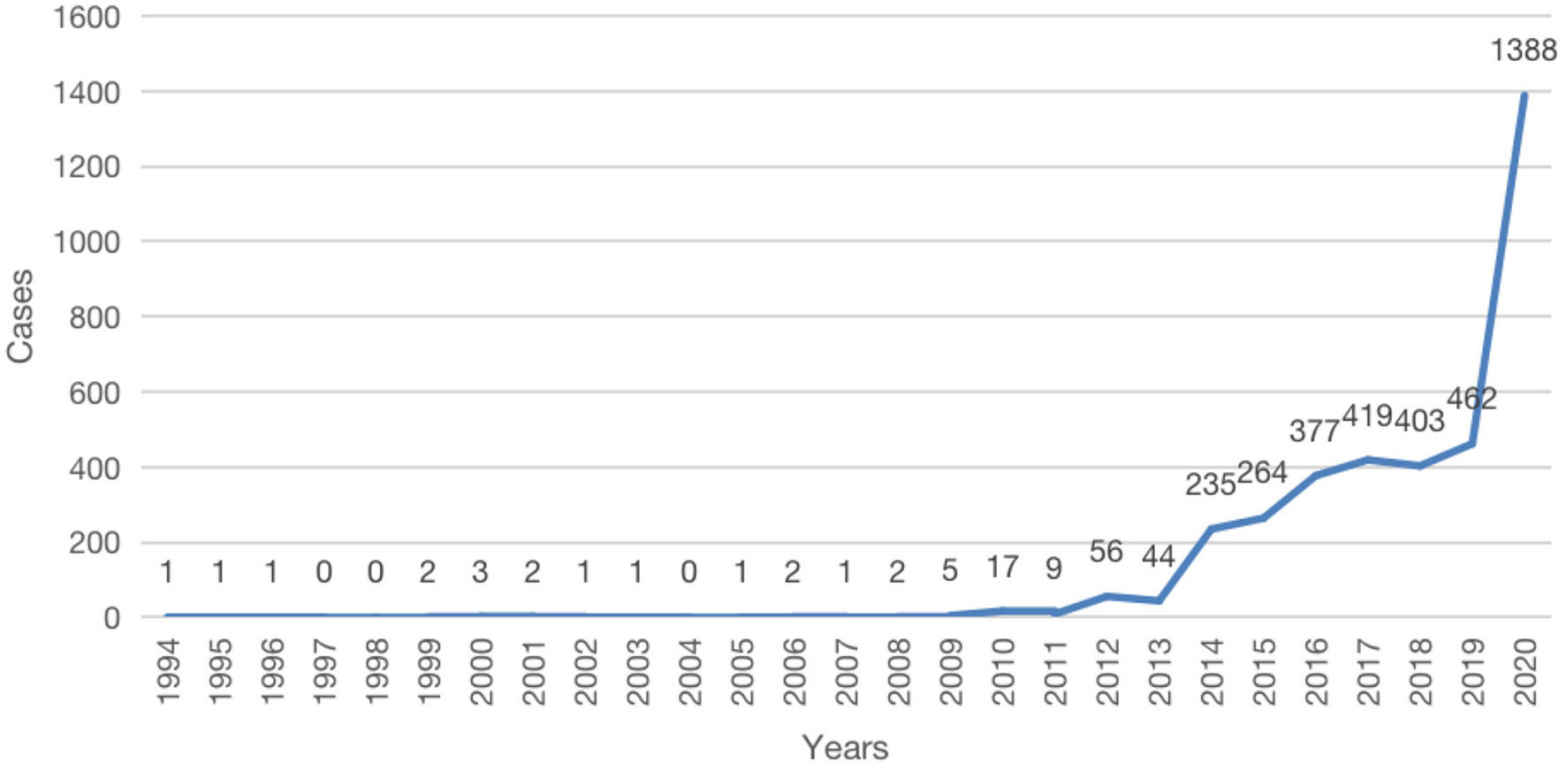
Time heard distribution.

Figure 1 shows that wildlife crime cases are concentrated from 2012 to 2020, with the highest number of wildlife crimes at 1,388 in 2020.

In 2000, the Supreme People's Court issued “Opinions on Several Issues Concerning the Concrete Application of Law in the Trial of Criminal Cases Involving Destruction of Wildlife Resources,” which reflected the awareness of wildlife protection at the national level. In 2003, China experienced a concentrated outbreak of SARS; thus, the Wild Animal Conservation Law was amended in 2004. The law experienced three amendments in 2009, 2016, and 2018. The three amendments indicate that national awareness of wildlife conservation has improved, but national awareness has not established a foundation for citizens to protect wildlife because wildlife crimes have not decreased. Although the National People's Congress (NPC), the decision-making organ in China, adopted the Interpretation of Articles 341 and 312 of the “Criminal Law of the People's Republic of China,” the number of crimes is still rising year-on-year across the country, and it peaked in 2020. The Standing Committee of the 13th NPC swiftly voted to pass a “Decision of the Standing Committee of the National People's Congress to Comprehensively Prohibit the Illegal Trade of Wild Animals, Break the Bad Habit of Excessive Consumption of Wild Animals, and Effectively Secure the Life and Health of the People.” The good news is that with this speedy decision passed by the NPC, the Chinese government launched a drive to fight wildlife crimes across China. Thus, there was a dramatic surge in the number of wildlife crimes in 2020. The coronavirus outbreak in 2020 witnesses that closer attention is given to this issue, and the situation is being alleviated around the country. It also reveals that the issues about wildlife markets and trade were overlooked by the central and local governments. The expanding market demand for wild animals due to China's economic boom, ingrained traditional culture of wildlife consumption and the desire to preserve the health of the people encourage consumer affluence. The increasingly greater law enforcement attention given by forest police and customs to wildlife offenses also made the number in 2020 very different from other years. However, social awareness of wildlife conservation is still relatively low, and the increasing number of wildlife crimes is the evidence for this.

### The Regions

**Figure 2:**
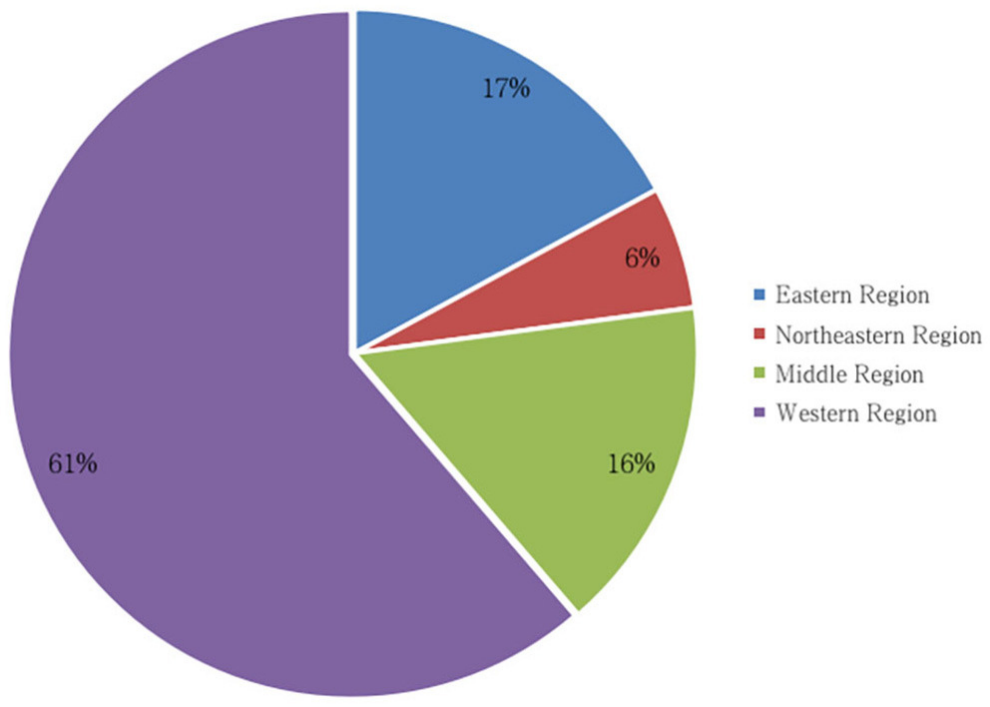
The regional distribution of wildlife crimes.

**Figure 3:**
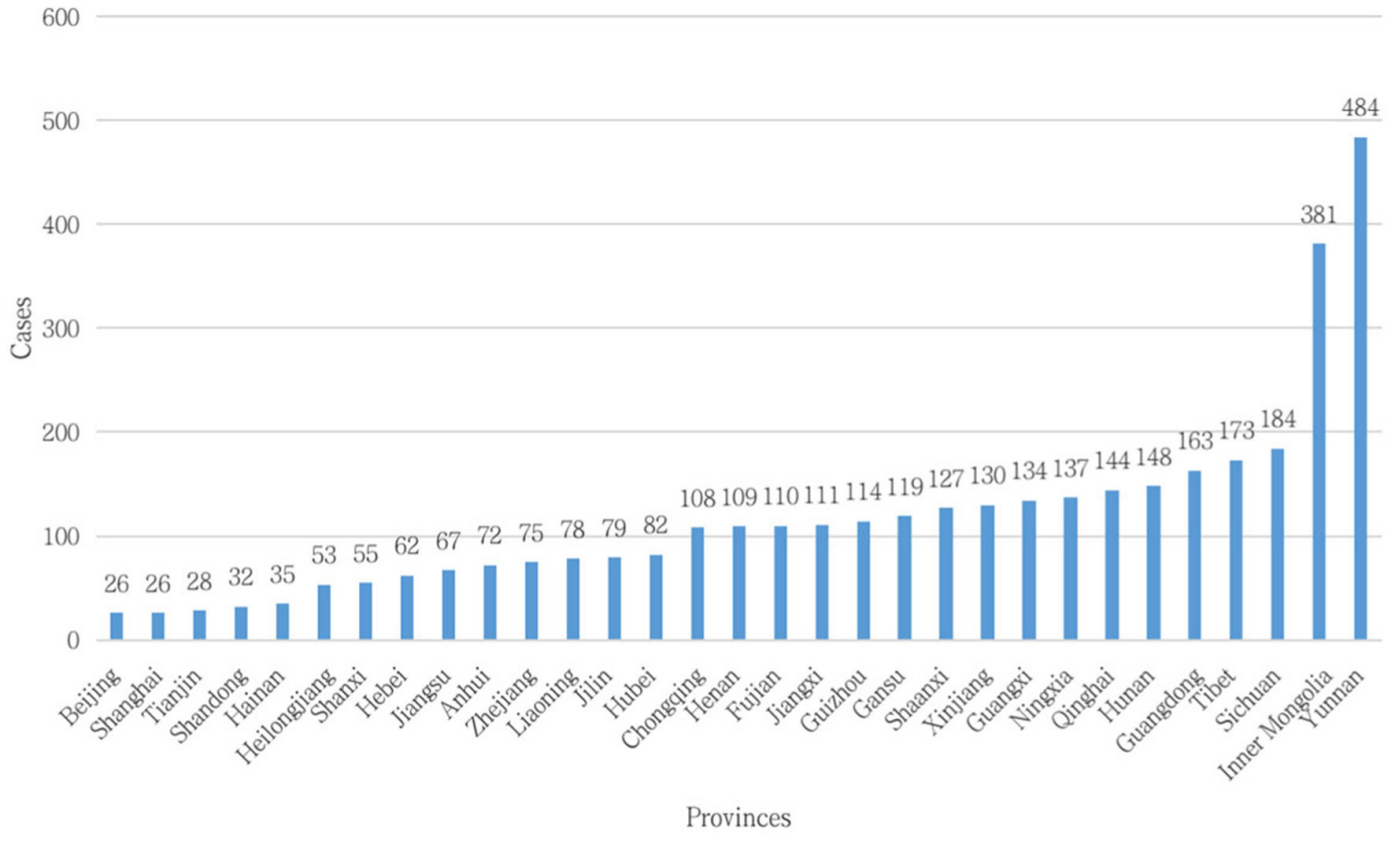
Provincial distribution of wildlife crimes.

The three provinces with the highest number of wildlife crimes (Figures 2, 3) are Yunnan, Inner Mongolia, and Sichuan Provinces. All are located in western China, which had 2,235 wildlife crimes, which constituted 61.30% of the country's total. The northeastern region had the lowest number of wildlife crimes at 210, or 5.76 %, while the middle and eastern regions had 15.82 and 17.11% wildlife crimes, respectively (Table 6).

**Table 6:** The regions of wildlife crimes.

**Region**	**Number**	**Proportion (%)**
Eastern region	624	17.11
Northeastern region	210	5.76
Middle region	577	15.82
Western region	2,235	61.30
Total	3,646	100

The western region ranks at the top in terms of nature reserves (Table 7), which accounts for 44.09% of them nationwide (9). The regions with the most nature reserves and the highest number of wildlife crimes are closely correlated. Rich wildlife resources are bestowed on people living in the western area, which objectively provides a breeding ground for wildlife crimes. Additionally, the western region is not economically developed compared with other regions nationally. Furthermore, the western region has a lower economic capacity. As a result, stimulated by economic incentives, crimes involving rare and endangered wild animals with high economic value are more rampant in this area. At the same time, the educational level is lower than that of the developed areas in eastern and middle China, and this area has underdeveloped educational resources; therefore, the education level is lower on average. Consequently, the awareness of wildlife protection is generally weaker.

**Table 7:** The distribution of nature reserves in China (8).

**Region**	**Number**	**Proportion (%)**
Eastern region	83	17.51
Northeastern region	92	19.41
Middle region	90	18.99
Western region	209	44.09
Total	474	100

## Discussion

### The Purpose of the Legislation and Legal Principle: Relationships Between Ecological Economic Ethics and Relative Laws

Human practice is generally self-conscious and purposeful. Marx argued that an end cannot be an end if it is not particular, just as an action is meaningless if it has no purpose (4). Legislation practice also follows this inherent rule. Legislation is a conscious activity that entails a certain purpose. Serving as the law's soul and guidance, the purpose of legislation is reflected in the design of the legal system. The purpose of legislation not only embodies the value of all legal provisions and objectives (10) but also restrains the legislature from acting beyond its authority, balances the social reality and different interests, and lays the foundation for the judiciary to interpret the spirit of the law and to judge cases accordingly. Therefore, the purpose of legislation is an important criterion for evaluating the quality of the legislation, and the realization of the purpose of the legislation must be considered when evaluating the implementation of laws.

The term “legal principle” refers to comprehensive principles and standards that can serve as the foundation or source for legal rules (11). According to Hart, legal principles are considered to involve some purpose, goal, entitlement, or value and are regarded as desirable to maintain or to adhere to. Therefore, legal principles not only provide an explanation or rationale for the rules that exemplify them but also contribute to their justification (12). In countries with written law, including China, principles of law are usually expressed by the legislature in the law, its interpretation or its enactment. Principles of law embody the guiding philosophy and values of the law.

#### Relations Between the Environmental Protection Law and Wild Animal Conservation Law

The “Environmental Protection Law of the People's Republic of China” (hereinafter referred to as the Environmental Protection Law) is the governing ecological environmental protection law in China. The “Wild Animal Conservation Law of the People's Republic of China” (hereinafter referred to as the Wild Animal Conservation Law) serves as the basic law for wildlife protection. The purpose of the two comprehensive laws can be divided into two levels of legislation. The first level has a general purpose; it potentially shapes people's attitude, choice and estimation of the law and enables members of society in general to participate in legislative activity to showcase their consensus on the law and legal values. Thus, it is possible to select, evaluate, and agree with legislation (13). Regarding this level, the legislative purpose of the Environmental Protection Law is of a higher order and encompasses diversified legislative purposes, such as environmental protection, pollution prevention and control, public health protection, the promotion of ecological civilization, and the development of economic society. The second level is the legislative purpose of specific legal sectors, which is determined by the specific function and role of different sectoral laws in adjusting different social relations. The purpose of legislation evolves as the social background changes and members of society upgrade their legal values. The Wild Animal Conservation Law was enacted in 1988 and was amended in 2004, 2009, 2016, and 2018. Its legislative purpose in 1988, 2004, and 2009 was to protect, rescue, and conserve rare and endangered species, to protect, develop, and rationally use wildlife resources, and to maintain the ecological environment balance. In the 2016 and 2018 versions, the purpose changed to protecting wild animals, rescuing rare and endangered wildlife, maintaining biodiversity and ecological balance, and promoting ecological civilization. The expression “protect, develop, and rationally use wildlife resources” was deleted from the legislative purpose in 2016 and 2018, and “maintain biodiversity” and “promote ecological civilization” were added. The ecological meanings of the purpose of the law were deepened. Accordingly, the legislative principles were revised from “strengthening resource protection, domesticating and breeding wild animals, and rationally developing and utilizing wild resources” to “prioritizing protection, utilization and strictly supervising” wildlife resources, with the additional connotation of protection and supervision.

At the same time, the scope of wild animals referred to in the Wild Animal Conservation Law was changed from “rare and endangered animals with economic and scientific value” to “rare and endangered animals with ecological, scientific and social value.” Although the focus changed from “economic value” to “ecological and social value,” the scope of protection is still classified by the values of animals' scarcity and availability (14).

The Environmental Protection Law, as the basic law in the field of environmental resource protection, still insists on two goals: environmental protection and economic social development. On the one hand, people relentlessly pursue rapid economic growth and continuously accumulate material wealth. On the other hand, people fear that the economy-centered model will become a burden on ecology and society. This anxiety showcases a contradiction between the excessive speed of development and the pressure of environmental burdens (15) and reflects a reluctant concession to the priority of economic development. It is not essentially ecological. Although the purpose, legislative principles and scope of protection of the Wild Animal Conservation Law have given way to some extent to protect wildlife, the substance and core are still rooted in the utilization and management of wildlife resources. This means that the law betrays its legislative purpose and principles (16). The Wild Animal Conservation Law, which focuses on the economic end, does not fully match its ecological ethical needs. A large part of the Wild Animal Conservation Law is devoted to wildlife utilization. It is still based on economic thinking (14), which indicates that the wildlife resource value remains unchanged (17). Additionally, the amended versions of the “Wild Animal Conservation Law” do not cover the prevention and control of major public health risks and lack consideration of public health and hygiene.

Therefore, the two basic laws in environmental and wildlife protection both fail to include the value of wildlife protection and the consideration of the value of public health and ecological ethics. Instead, people fall prey to utilitarian economic value.

#### Relations Between the Criminal Law and Wild Animal Conservation Law

The “Criminal Law of the People's Republic of China” (hereinafter referred to as the Criminal Law) was originally established to combat crimes, protect citizens' lives and property, and maintain social and economic order. Subsequently, current wildlife-related offenses under the Criminal Law have focused on punishing the disruption of the economic order and protecting state property while preventing crimes. Thus, wildlife crime provisions do not embrace the discipline of ecological ethical misconduct and concentrate only on the economic value of wildlife resources. Regarding the provisions for wildlife crimes and adjudication practices, the configurations of the penalties are inconsistent: the types of wildlife crime punishment are relatively homogeneous, and some misconduct is not addressed by the Criminal Law; thus, the cost of committing the offense is low. These findings indicate that the penalties have an insufficient deterrent effect on perpetrators and fail to effectively reflect the punitive effect of this law on wildlife crimes and the role of the law in improving the ethical quality of the ecological economy (18). Regarding the subjective aspects of the crime, wildlife crime offenders have both negligence and intentional considerations, but the common feature is that they view economic interests as the most important incentive; therefore, the concept of ecological morality is not formed subconsciously. Concerning the object aspect of the crime, the wildlife crime provisions in the Criminal Law describe violations of property interests and the national wildlife protection order, but more attention is given to the stability and orderliness of property interests. From an objective viewpoint, the provisions for wildlife crime discuss social harm and consequences. Articles 341 and 151 of the Criminal Law make the “seriousness of circumstances” a condition for aggravating the statutory punishment for wildlife protection. The “Judicial Interpretation of Criminal Cases Involving Wild Animals” provides for “serious circumstances” and “particularly serious circumstances,” and it rules that for the serious cases that include excessive wild animals beyond regulations and seriously harmful means, high economic value can be defined. This judicial interpretation provides for “aggravated circumstances” and “particularly aggravated circumstances.” However, it is unclear how to understand the criteria in the addenda tables for interpreting the terms of “illegal hunting, killing, purchase, transportation, and sale of rare and endangered wild animals,” “serious circumstances,” “particularly serious circumstances,” and “quantity criteria.” Are these criteria interpreted in accordance with ecological science or laws? Is the clause the tool for judges to abuse their discretionary power? These issues are unclear. Therefore, the supporting judicial interpretation for punishing wildlife crimes needs to be discussed further.

### Wildlife Crimes and Ecological Economic Ethics

As we discussed before, the purpose of legislation serves the needs of certain guidelines and values. However, it is so abstract that it must be interpreted into specific rules or articles. We discuss the relations between wildlife crimes and ecological economic ethics. In a sense, the article crystallizes the purposes of the legislation.

#### The Notion: Ecological Economic Ethics

A legal system must exhibit specific conformity with morality or justice or rest on a widely diffused conviction so that there is a moral obligation to obey it. That is, it follows that the criteria of the legal validity of particular laws in a legal system must include, tacitly if not explicitly, a reference to morality or justice (13). As we mentioned before, the Standing Committee of the 13th NPC passed a decision. However, the spokesman also confessed that this move was temporary and that the comprehensive revision of wildlife protection was a long-term goal (19). At the same time, some cities and provinces in China have introduced local regulations to combat the indiscriminate consumption of wild animals; however, supporting legislation is still pending. Although the law is not omnipotent, and legislation is not the sole solution for wildlife crimes, effectively implemented and enforced laws are those consistent with or similar to prevailing customs (20). Thus, without a shift in awareness about wildlife, legislation is still bounded by the superficial level of economic value. Even though wildlife legislation is sophisticated and well-designed, authorities cannot expect it to be automatically accepted by the people and transferred into actions by the public. Therefore, the legal system must combine the law with the conscious acceptance of people's moral sentiments to produce a solid social foundation for the legal system.

It is suggested that given the subjective factors of wildlife crimes, offenders only consider economic interests and have weaker ecological ethical awareness. Regarding the objective consideration of wildlife crimes, the original aim of establishing wildlife crime clauses is to maintain the economic order. The scope of wildlife animals protected by the law is also limited to the animals that are of use or that have economic value; however, animals with little economic value but great ecological value are not on the protected list. Considering the incidence and sentencing of wildlife crimes, although the Wild Animal Conservation Law has been amended several times, its amendments have not produced a positive impact on the deterrence of wildlife crimes as expected. In judicial practice, this can result from a dismissive ecological ethic and lighter punishment or even no punishment since the judges focus excessively on the necessity principle of criminal law. This is demonstrated by the fact that illegal hunting actions are punished by fines but not by harsher sentences; there is a high proportion of suspended sentences, and the average sentence is short. These situations illustrate the limitations of using economics as a standard to judge wildlife crimes. Therefore, it is necessary to introduce ecological economic ethics to compensate for the shortcomings caused by prioritizing economics.

Given the large number of issues posed by prioritizing economic interests, calls for ecological ethics and ecological morality are snowballing, and ecological economic ethics is in the spotlight. Leopold expands the content of ethics from studying the relationships among people and between people and society to the relationship between people and the land (21). He notes the limitations of traditional ethics (22) and proposes the concept of “land ethics,” which is the prototype of ecological ethics. Holmes Rolston develops the doctrine of land ethics and proposes considering a fundamental, natural sense of environmental ethics (23). However, in the current context of anthropocentric ecology, solely emphasizing ecological ethical values may ignore reality because economic activity is the basis of human existence. Living standards will regress if people overlook economic development. This ideology can be echoed by law, which regulates the interrelationships among human beings. Without this consensus, the ideology that protects a pure environment with no human participation cannot be accepted, recognized, or protected by law (24). Again, it is necessary to introduce the economic-centered development model and choose a sustainable ecological economic path.

Ecological economic ethics regulates the economy with ecological ethics and explores the rationality of economic development, which means that in traditional economic practice, ecological elements are included in the indicators of economic evaluation and ethical considerations. The rationality of economic behavior is related to whether it meets the requirements of natural law and ecological environmental protection. With the integration of ethics, ecology, and economics (25), ecological economic ethics refers to people obeying ethical norms, building ethical relationships and performing ethical practices to consciously reconcile and balance economic construction, social development and environmental protection in economic society. Ultimately, people can unite and balance social, economic, and ecological benefits. Ecological economic ethics justifies the promotion of the ecological economy and a sustainable development path from an ethical perspective, which paves the way for a moral order or moral climate (26). According to Leopold, human actions can be explained as the result of an aggregation of individual actions that originate from the inner will. In addition to their hunting and economic value, wild animals have potential significance for all humanity that most people are unaware of (22). One of the most important reasons for the persistence of wildlife crimes is that people lack ecological economic ethics, which entails powerful moral means because laws are incapable of solving the problem. People's obsession with money should be upgraded to ecological economic awareness to thus create an ecological economic atmosphere in society.

#### Ecological Economic Ethics Model of Wildlife Crimes

Ecological economic ethics plays two roles: to provide the necessary moral defense for the development of the ecological economy and to create the necessary moral order or moral atmosphere. Therefore, the “internalization” of eco-economic ethics can provide a new perspective to seek a more universal and inherent moral mechanism and perspective for the “human-nature-human” behavior model. Ecological economic ethics can be divided into three parts, namely, awareness, rules and practices. To fulfill the mission of eco-economic ethics, it is necessary to go through the three levels of moral awareness, moral rules and moral practice. First, moral awareness is needed to establish the ideology and cognition of ecological ethics and to clarify the boundary between morality and immorality. Second, internalized or externalized rules are used to embody and express ecological ethics. Finally, practice can promote the guidance of economic behavior through the operation of ecological ethics rules to form a benign interaction. Thus, the connection between wildlife crimes and ecological economic ethics must be considered. First, as one of the extreme behaviors of polluting the environment or causing great ecological damage, how can the moral failure of wildlife crimes be reflected? Is it a subjective consciousness or an objective behavior? Is it, and how should it be judged from a value standard? Second, can the penalties of wildlife crimes meet the demand of eco-economic ethics? That is, can the judicial interpretation of wildlife crimes improve the quality of eco-economic ethics?

Specifically, ecological economic ethics can be divided into three parts, specifically, ethical awareness, ethical rules, and ethical practice. A model can bridge the gap between the four constitutive elements of wildlife crimes (this paper adopts the “Four Constitutive Elements Theory,” i.e., the subject, the subjective, the object and the objective) and the three levels of ecological economic ethics (awareness, rules and practice) (18). The model is shown in Table 8.

**Table 8:** Ecological economic ethics model of wildlife crimes (18).

**Constitutive elements**	**Ecological economic ethics model**	**Measures**	**Consequences**
The subject	Awareness	Strong or weak social awareness	If the subject is a unit, then its social awareness is strong. If the subject is a natural person, then its social awareness is weak.
The subjective	Awareness	Value judgment of awareness	If the offenders are criminally negligent, then they have strong social awareness with a neutral attitude toward the crime. If the offenders are criminally intentional, then they have weak social awareness with malice.
The object	The static rules	How to protect the ecological ethic relationship	If it relies on the social relations run by public power, then the standard of judgment is high, but it is easy to judge. If it relies on the social relations protected by private law, then the standard of judgment is low, but it is difficult to judge.
The objective	The dynamic rules	Value ranking in measures	If it takes an economic value as the judgment, then it is easy to judge and achieve. If it takes an ecological value as the judgment, then it is difficult to judge and achieve.
The cases	Practices	Width of ethic misconduct	If the misconduct is wide, then the cases are excessive. If not, then the cases are less quantitative.
Sentencing	Practices	Depth of ethical misconduct	If the level of misconduct is deep, then the sentencing is heavy. If not, then the sentencing is light.

From the subject view of wildlife crimes, these crimes are mainly committed by natural persons, which indicates that the parties are less socially conscious. From the subjective viewpoint of wildlife crimes, the social awareness of the people who commit negligent crimes is neither good nor bad but socially conscious. The offenders of intentional wildlife crimes have malice and weak social awareness. From the perspective of the object, wildlife crimes destroy the social relations that operate through private rights; thus, they are difficult to judge. From the perspective of the objective, wildlife crimes center on economic value, which is easy to judge and achieve. The high incidence and excessive practices of wildlife crimes demonstrate that offenders defy ecological economic ethics. This high incidence and these excessive practices show the deep misconduct of ecological and economic ethics. However, at the same time, we should hold a dynamic and dialectical view about the volume of cases. A high number of cases is generally considered to be more serious moral misconduct. However, from the perspective of adjudication, a high number of cases may also mean that certain provisions that correspond to the crime are invoked more than other provisions to thus become “popular” provisions, which increases the frequency of punishment. For example, after the outbreak of the 2020 epidemic, the surge in the number of wildlife cases illustrates the increase in punished wildlife misconduct brought about by the enactment of rules created with a strong awareness to thus enable more moral misconduct to be deterred or punished by law.

Different dimensions of wildlife crimes echo different ecological economic ethical dimensions. The details are as follows.

As shown in Table 9, although all of the crimes are committed by natural persons, the corresponding ecological economic ethics dimensions of different wildlife crimes vary, such as the “illegal hunting and killing of rare and endangered wild animals” and “illegal purchase, transportation, and sale of rare and endangered wild animals and their manufactured products.” The two crimes are abundant in real life because of weak social consciousness, such as the ecologically friendly and green-environment awareness of the parties involved. Second, the offense is easy to judge since it focuses on economic values. Conversely, although offenders of the illegal hunting and smuggling of rare animals and the illegal transportation and sale of rare and endangered wild animals have weak social awareness, they destroy administrative licenses such as hunting licenses and chartered hunting licenses. The crime of smuggling rare animals and precious animal products infringes on the national trade management system (27). As a result, it is easy to identify and judge.

**Table 9:** Ecological economic ethics dimensions of wildlife crimes (18).

**Clauses**	**Crimes**	**Ecological economic ethics**					
		**Awareness**		**The static rules**	**Practice**		**The dynamic rules**
		**The subject**	**The subjective**	**The objects**	**The cases**	**Sentencing**	**The objective**
Article 341I	Illegal hunting and killing of rare and endangered wild animals	Weak social awareness	Uncertain	It is easy to judge and achieve.	The cases are excessive.	Sentencing is light.	It is difficult to judge and achieve.
Article 341 I	Illegal purchase, transportation, and sale of rare and endangered wild animals and their manufactured products						
Article 341 II	Illegal hunting				The cases are less quantitative.		It is easy to judge and achieve.
Article 151 III	Smuggling of rare animals, illegal transportation, and sale of rare and endangered wild animals						

Accordingly, eco-economic ethics are rarely seen in wildlife crimes. The reasons are as follows. The interests protected by wildlife crimes are relatively scattered and fragmented, and they fail to cover all aspects of the ecosystem and establish an overall and systematic meaning of eco-economic ethics. Second, most of these interests reflect or emphasize economic value instead of ecological value. From an internal perspective, although the criminal composition of wildlife crimes and eco-economic ethics are closely related, the corresponding relationships between different variables are not consistent and show a non-linear distribution.

## Suggestions

Dangerous zoonotic diseases are not only highly contagious but also highly lethal. Studies indicate that 70% of new diseases will originate from wild animals (28). The widespread lack of ecological economic ethics has led people to focus on the economic value of wildlife and to ignore its health risks. People should bear in mind the separation of the economic and ecological value of wild animals, whose neglect could eventually lead to a public health crisis across the globe. Therefore, promoting ecological economic ethics as a guide, implementing the notion of public health and improving relevant legislation are the keys to safeguarding human health and preventing public health crises.

The penal sanction in the Criminal Law, as the final defense of the legal system, is the most severe sanction. The Criminal Law is designed to provide final protection (24) for the legal interests that are established and protected by preceding laws in the overall legal order. The improvement of crime prevention mechanisms lies not only in amending criminal laws but also in observing the laws that precede criminal laws to constitute systematic legislation for wildlife protection. Furthermore, although it marks the cornerstone of wildlife protection law, the Wild Animal Conservation Law includes a narrow range of wildlife that should be protected; consequently, the supporting regulation, the List of Key Protected Wild Animals, does not cover the wild animals that are of ecological value to the environment. The scope of “protected wild animals” in this list should be expanded to include more wild animals. As a basic law in environmental protection, the Environmental Protection Law has established the “protection of public health” among its legislative purposes; article 39 provides for an environmental and health monitoring, investigation and risk assessment system, and incentive measures, while article 47 establishes an emergency and warning system for the environment and public health. In contrast, as an indispensable part of the legal framework for environmental protection and natural resources, the Wild Animal Conservation Law does not reflect the values of public health and hygiene and fails to protect public health as it should. For this reason, the legal system should be equipped with content related to public health and should integrate public health content into the different legal sectors and dimensions. To coordinate with different laws, a system to protect public health can be formed with close institutional links and reasonable structural arrangements to maintain public health security.

Five points should be discussed.

1. Support legal obligation mechanism. Under the principle of punishment, the supporting legal obligation mechanism plays a critical role in combating wildlife crimes. Although the severity of the penalty and the intensity of combating crime may not be coincidentally correlated, according to the principle of fitting the punishment to the crime, the severity of the penalty reflects the level of harm and awareness of the crime (29). For wildlife crimes, the average sentencing term is <3 years, and a lesser term is likely less costly for the offender, which makes it difficult to renew the wildlife conservation order. Therefore, it is necessary to impose more obligations by increasing penalties. The punishment standard that solely depends on the economic value of wild animals and their products should be optimized by taking into account ecological economic ethics. The legal obligation for the consumption of wild animals can be explicitly stipulated in article 337 of the Criminal Law. People can form an expectation of the wildlife protection order through the deterrent effect of criminal law, which can effectively deter wildlife crimes.

2. Implement the precautionary principle. The “precautionary principle” was established in the “Rio Declaration on Environment and Development”: countries should extensively use precautionary measures to protect the environment according to their capabilities. Where there is a threat of serious or irreversible damage, countries are not allowed to postpone precautionary measures with the excuse that there is a lack of scientific and sufficient evidence (30). Humanity has also learned a lesson from this epidemic. Without risk prevention mechanisms in place, a highly contagious and devastating disease can create a public health and safety crisis whose consequences are difficult to predict. Recently, science has been incapable of studying the uncertainty between viruses in wildlife and zoonotic infectious diseases; therefore, ecological ethics and an understanding of the relationship between human beings and nature are badly needed. It is necessary to eliminate the indiscriminate consumption of wild animals, such as by creating an eating blacklist that restricts wildlife consumption, adding preventive mechanisms for public health, and using risk contingency plans in the Wild Animal Conservation Law to deepen the implementation of the precautionary principle. Additionally, as a supplement to the laws, an article should be included in the Criminal Law to prevent people from consuming wild animals.

3. Improve public participation in legislation. Citizens, or the public, practice democratic decision making, participate in social life, and enjoy environmental rights. These activities play an important role in standing up for the right to make laws, impeach authorities, accuse, and supervise in their interests. Therefore, this principle is an essential element of environmental governance. The implementation of this principle contributes to safeguarding citizens' environmental rights, improving the efficiency of environmental governance, and realizing environmental democracy and justice. We believe that public and social organizations, including environmental and animal protection organizations, should play their full role in environmental protection and wildlife conservation, while the government should improve the participation of citizens and social organizations in the prevention and control of major public health risks and in legislative procedures.

4. Break the bad habit of eating wild animals. First, we should keep in mind the idea that wild animals are an indispensable part of the environment that maintain biodiversity; thus, they are beneficial to the ecosystem. In a sense, this point has been emphasized numerous times, but people still have narrow-minded perceptions. Second, wild animals may carry diseases that can cause zoonosis, as demonstrated by great epidemics in research and human history. Preserving one's health is currently trendy. Influenced by traditional Chinese medicine and overstated rumors about the benefits of wildlife, unreasonable people rush to the wildlife market and believe that wild animals can cure rare illnesses and even save people's lives. Modern medicine has proven that wild animals' bones or organs fail to perform this function. Finally, wild animals are the best choice for a loss of appetite. Our predecessors have helped us choose tasty food instead.

5. Strengthen the guidance and education function of schools. Contemporary education is centered on economic supremacy. The renowned ecological economist John B. Cobb Jr. reflected on and criticized the role of schools, and he noted that the purpose of schools currently is to serve the economy, which betrays the aim of serving an ecological civilization and the common welfare of human beings and nature (31). Therefore, legislation can encourage schools to take on the guidance and education of ecological economic ethics by cultivating a climate of awareness of ecological economic ethics, strengthening beliefs in ecological economic ethics, and developing environmentally friendly behaviors. Schools should encourage students to break the illicit trend of consuming wild animals due to blind regimens and hunting and should cultivate positive feelings for animals and the environment. In this way, sustainable development and a green economy can be adopted by students, and schools can effectively play a role in guiding and educating students.

## Conclusion

This paper analyzes the subject and subjective factors, time, areas, sentencing term, and suspended and additional sentences to reveal the lack of ecological economic ethics in the legislative purposes and principles of the Environmental Protection Law, the Wild Animal Conservation Law, and the Criminal Law. By establishing a model of ecological economic ethics, we find that the social awareness of wildlife crime is weak. From the subject view, wildlife crimes are mainly committed by natural persons, which indicates that the parties are less socially conscious and that their social awareness is neither good nor bad. From the perspective of the object, wildlife crimes destroy the social relations that operate according to private rights; therefore, they are difficult to judge. From the perspective of the objective, wildlife crime offenses center on economic value, and they are easy to judge and achieve. The high incidence and excessive practices of wildlife crimes demonstrate that offenders defy ecological economic ethics. The high incidence and practices indicate deep misconduct. The corresponding ecological economic ethics dimensions of different wildlife crimes vary, such as those for the “illegal hunting and killing of rare and endangered wild animals” and the “illegal purchase, transportation, and sale of rare and endangered wild animals and their manufactured products.” The culprit of these two crimes is weak social consciousness, such as the ecologically friendliness and green-environment awareness of the parties involved. These factors show that the existing judicial and legal systems fail to fully embody the value of ecological economic ethics and that legislation related to wildlife protection that values ecological economic ethics is not yet in place; the law is still at the level of a tool rather than reflecting the ultimate value of ecological economic ethics. Therefore, this paper advocates for the deepening of ecological economic ethics consciousness and the establishment of a public health system. Ultimately, we will comprehensively establish a legislative system for ecological economic ethical value and public health.

## Data Availability Statement

The original contributions presented in the study are included in the article/supplementary material, further inquiries can be directed to the corresponding author.

## Author Contributions

ZZ contributed to the study design, including the conceptualization and methodology, prepared datasets, and promoted project administration. YZ contributed to the resources, data analysis, and original draft preparation. DX contributed to the study's organization, identified the research methods, and performed the review and editing of the manuscript. All authors read and approved the final manuscript.

## Funding

This study was financially supported by the Major Program of the National Social Science Foundation of China: Research on the legal innovation of ecological environment supervision system in the new era (18 VSJ039).

## Conflict of Interest

The authors declare that the research was conducted in the absence of any commercial or financial relationships that could be construed as a potential conflict of interest.

## Publisher's Note

All claims expressed in this article are solely those of the authors and do not necessarily represent those of their affiliated organizations, or those of the publisher, the editors and the reviewers. Any product that may be evaluated in this article, or claim that may be made by its manufacturer, is not guaranteed or endorsed by the publisher.
